# Expanding the Phenotypic Spectrum of Pathogenic *KIAA0586* Variants: From Joubert Syndrome to Hydrolethalus Syndrome

**DOI:** 10.3390/ijms25147900

**Published:** 2024-07-19

**Authors:** Desirée Deconte, Bruna Lixinski Diniz, Jéssica K. Hartmann, Mateus A. de Souza, Laira F. F. Zottis, Paulo Ricardo Gazzola Zen, Rafael F. M. Rosa, Marilu Fiegenbaum

**Affiliations:** 1 Programa de Pós-Graduação em Patologia, Universidade Federal de Ciências da Saúde de Porto Alegre, Porto Alegre 90050-170, Brazil; desideconte@gmail.com (D.D.); bruldiniz@gmail.com (B.L.D.); 2 Faculty of Medicine, Universidade Federal de Ciências da Saúde de Porto Alegre, Porto Alegre 90050-170, Brazil; jekarineh1997@gmail.com (J.K.H.); mateuss@ufcspa.edu.br (M.A.d.S.); lairazottis@gmail.com (L.F.F.Z.); 3 Departamento de Clínica Médica, Universidade Federal de Ciências da Saúde de Porto Alegre, Porto Alegre 90050-170, Brazil; paulozen@ufcspa.edu.br (P.R.G.Z.);; 4 Departamento de Ciências Básicas da Saúde, Universidade Federal de Ciências da Saúde de Porto Alegre, Porto Alegre 90050-170, Brazil

**Keywords:** ciliopathies, hydrolethalus syndrome, *KIAA0586*, pathogenic variant, inheritance, variable expressivity

## Abstract

*KIAA0586* variants have been associated with a wide range of ciliopathies, mainly Joubert syndrome (JS, OMIM #616490) and short-rib thoracic dysplasia syndrome (SRTD, OMIM #616546). However, the hypothesis that this gene is involved with hydrolethalus syndrome (HSL, OMIM #614120) and orofaciodigital syndrome IV (OMIM #258860) has already been raised. Ciliopathies’ clinical features are often overlapped despite differing in phenotype severity. Besides *KIAA0586*, *HYLS1* and *KIF7* are also known for being causative of ciliopathies, indicating that all three genes may have similar or converging genomic pathways. Overall, the genotypic and phenotypic spectrum of ciliopathies becomes wider and conflicting while more and more new variants are added to this group of disorders’ molecular pot. In this case report we discuss the first Brazilian individual clinically diagnosed with hydrolethalus syndrome and molecular findings that demonstrate the role of *KIAA0586* as a causative gene of a group of genetic disorders. Also, recent reports on individuals with intronic and exonic variants combined leading to ciliopathies support our patient’s molecular diagnosis. At the same time, we discuss variable expressivity and overlapping features in ciliopathies.

## 1. Introduction

The cilia are an essential component of signal transduction during embryonic development involved in cellular detection and the management of external signals. Dysfunctional cilia are often associated with ciliopathies, a heterogeneous group of genetic diseases that manifest during childhood and adolescence [1,2,3]. The majority of syndromes related to genes involved in ciliary function are monogenic and have an autosomal recessive trait. Although the phenotypic spectrum is known to be wide, the main clinical features observed in ciliopathies encompass retinal degeneration, renal disease, skeletal malformations, brain anomalies, and neurological impairment [1,2,3,4,5,6].

Genes related to ciliopathies are called *ciliary genes*, and over 800 genes have already been identified as involved in the ciliary–centrosome function [7]. Among the ciliary genes, *KIAA0586* (OMIM #610178, 14q23.1), the human ortholog of mouse and chicken *TALPID3*, encodes a conserved centrosomal protein required for sonic hedgehog signaling (Shh), vertebrate development, and ciliogenesis [8,9,10]. Previous animal model studies showed that the *KIAA0586* knockdown phenotype is associated with severe ciliogenesis impairment and Shh disruption [11,12]. Primary cilia have an essential role in sonic hedgehog signaling in mammals, and their function is essential for correct embryonic formation. As a result, variants in ciliary genes like *KIAA0586* are associated with severe clinical consequences [11,12].

Pathogenic variants in *KIAA0586* are often associated with a wide range of ciliopathies, such as Joubert syndrome (JS, OMIM #616490) and short-rib thoracic dysplasia syndrome (SRTD, OMIM #616546), and the hypothesis of this gene being involved with hydrolethalus syndrome (HSL, OMIM #614120) and orofaciodigital syndrome IV (OMIM #258860) has been raised in the literature [4,5,6,8,13]. Although the severity of ciliopathies may differ, a variety of clinical manifestations are frequently overlapped. Moreover, these diseases have molecular heterogeneity, with several genes being associated with one or more ciliopathies [4,14]. Therefore, the etiology of these syndromes as well as the role of the involved genes are yet to be elucidated to understand the genotype–phenotype correlation behind ciliopathies disorders [14].

Here we report on the first Brazilian individual clinically diagnosed with hydrolethalus syndrome and molecular findings that instigate the discussion on the role of *KIAA0586* as a causative gene of ciliopathies. Also, we provided an overview of the wide phenotype range in the ciliopathies spectrum as well as the overlapping genes between them by gathering previous cases reported in the literature and proposing whether gene expression variability among ciliopathies is a possibility.

## 2. Results

### 2.1. Clinical Findings

The proband was the first child of young and non-consanguineous parents with no similar cases in the family, although the father’s sister was described as being born with clubfoot. Both parents have German ancestry. A previous history of pregnancy loss was denied.

The mother had two episodes of bleeding in the third month of pregnancy. She used piperidolate hydrochloride and progesterone but denied smoking or any drug use during gestation. In the second trimester, ultrasound and magnetic resonance imaging (MRI) identified Dandy–Walker malformation, corpus callosum agenesis, microphthalmia, reduced chest diameter, short limbs, increased amount of amniotic fluid (polyhydramnios), and a retrovesical cystic mass, which, postnatally, was found to be a didelphic uterus with hydrometrocolpos; the fetal echocardiography was normal. The parents chose not to perform fetal karyotyping by amniocentesis.

The proband, female, was born prematurely at 35 weeks and 2 days by cesarean section, weighing 2649 g (percentile 70) and measuring 46 cm (percentile 53), with a head circumference of 33 cm (percentile 83) and Apgar score of three at the first minute and five at the fifth minute. She presented respiratory distress and needed to be placed on mechanical ventilation. During the physical examination, a large anterior fontanelle, deep-set eyes with an impression of microphthalmia, low and broad nasal root, median cleft lip, cleft palate, micrognathia, hypoplastic and malformed tongue, dysplastic, small and low-set ears with a preauricular pit on the left, short neck with redundant skin, narrow thorax, postaxial polydactyly of hands, wrists arthrogryposis; camptodactyly of fingers, labia majora hypoplasia, anteriorly placed anus, and broad/bifid hallux (preaxial polydactyly) were noted. A brain ultrasound confirmed the findings observed during the prenatal period and identified the presence of dilated lateral ventricles. Abdominal/pelvic ultrasound showed pielocalicial and ureteral dilatation, as well as a didelphic uterus with hydrometrocolpos. The ophthalmologic assessment confirmed the apparent bilateral microphthalmia. A radiographic evaluation showed an elongated skull with a large posterior fossa, a small jaw, a narrow thorax with little expanded lungs and short ribs, wide iliac bones with a small sciatic notch, underdeveloped vertebrae, and humerus shortening. The child died within 25 days of life due to sudden respiratory dysfunction. A postnatal MRI could not be performed.

### 2.2. Genetic Analysis Results

Chromosomal microarray analysis revealed a deletion at chromosome 7q (71,869,384–76,820,289), a deletion at chromosome 14q (38,465,567–38,584,405), and two deletions at chromosome Xq (62,036,670–67,171,633; 71,632,324–78,216,024). Gene content for these deletions can be found in Supplementary Table S1. In addition, genes in the deleted regions were crossed with all ciliary genes described in the KEGG database to identify potential involvement in cilia function (Supplementary Table S2). At chromosome 7, two ciliary genes—*CLDN3* and *CLDN4*—were found to be deleted; however, neither of them justifies the clinical features presented in our case. Moreover, none of the alterations identified by CMA were associated with the proband phenotype. A pathogenic variant in *KIAA0586* was identified by NGS (NM_001244189.1:c.4018del, p.Glu1340ArgfsX10) in both the father’s and proband’s samples. This variant was not described in the literature. The mother sequence was normal. However, rtPCR analysis showed a probable intronic mutation at *KIAA0586* in the mother’s sample, which led to an aberrant transcript. This result suggests that the second causative variant may be a mutation not identified by NGS inherited from the mother. Although CMA results showed a deletion on chromosome 14, the deleted region does not comprise the *KIAA0586* nor any known gene (Supplementary Table S1).

Unfortunately, proband rtPCR could not be performed and the inheritance of the mother’s intronic variant was not confirmed. Hydrolethalus is an autosomal recessive syndrome, which implies that a variant in both alleles is to be expected to justify the lethal phenotype. Future research should consider intronic analysis before rejecting any autosomal recessive disease hypothesis or suspicion.

## 3. Discussion

Hydrolethalus syndrome (HLS) is a lethal genetic syndrome with an autosomal recessive trait characterized by the association of postaxial polydactyly and preaxial polydactyly of hands and feet, micrognathia, and hydrocephaly or anencephaly with a key-hole defect of the occipital bone [15]. Researchers have associated *HYLS1* and *KIF7* as key genes in this disorder [16,17]. The *HYLS1* gene showed alternative splicing, where the transcript was found to be present in multiple tissues during fetal development [18]. Also, an experiment with mice harboring the *HYLS1* variant showed that the animals die shortly after birth and exhibit developmental defects that resemble several manifestations of the human syndrome [19]. The *KIF7* gene, on the other hand, encodes a cilia-associated protein that regulates the hedgehog signaling pathway. In vivo genetic interaction studies indicated that the knockdown of *KIF7* could exacerbate the ciliopathy transcripts since KIF7 expression in cell lines causes defects in cilia formation and induces abnormal centrosome duplication [17,20]. However, the case reported in this paper differs from those already described, since the molecular finding on an HLS individual was on the *KIAA0586* gene.

Although *KIAA0586* is not the first candidate gene for HLS, it is associated with ciliopathies [6,8,21]. Ciliopathies have overlapping features, including renal and liver disorders, retinal degeneration, central nervous system defects, abnormal bone growth, and polydactyly [2,3]. Joubert syndrome was one of the first ciliopathy disorders recognized after the identification of the subcellular localizations and functions in the defective proteins [2,3,22]. JS phenotype is based on neurological features, abnormal eye movements, ataxia, cognitive impairment, and agenesis of the cerebellar vermis [23]. To date, several genes have been identified as causing the syndrome. However, many patients remain undiagnosed, suggesting that genetic heterogeneity may justify the reason why molecular diagnosis can be challenging [21]. Whole exome sequencing (WES) is preferable, owing to ciliopathies’ high diagnostic yield. In 2017, Vilboux et al. [24] suggested that if WES is not possible, then updated Joubert syndrome gene panels are preferable in most cases. In addition, in the absence of mainly phenotypic clues, prioritization of the most prevalent JS genes, including *KIAA0586*, may be reasonable. The mutation of known genes accounts for approximately half of the cases. Similar to our study, in 2015, Stephen et al. [25] identified a novel locus with a homozygous splice site mutation in *KIAA0586*, a known lethal ciliopathy locus in model organisms, in patients clinically diagnosed with JS. The truncating *KIAA0586* mutations observed may result in loss of gene function and consequently abnormal tissue polarity, centrosome length and orientation, and centriolar satellites.

**Figure 1:**
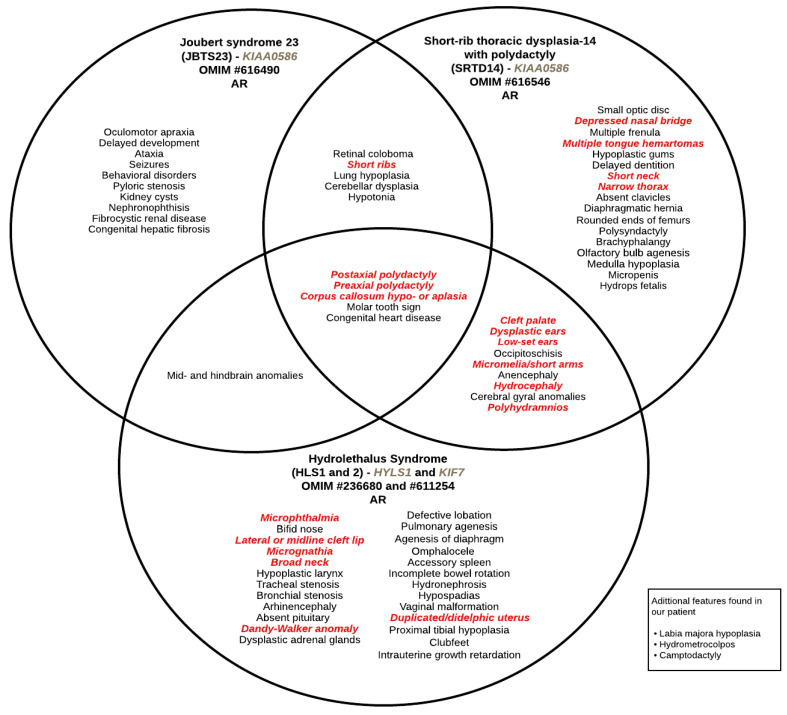
Venn diagram showing described overlapping clinical phenotypes between ciliopathies, as well as single syndrome features. Features written in red correspond to the ones found in our patient. AR: autosomal recessive.

The phenotype of KIAA0586-affected subjects may range from a hydrolethalus phenotype to SRTDS. In 2015, Alby et al. [4] identified homozygous nonsense and splicing *KIAA0586* mutations as responsible for lethal ciliopathies in patients with similar phenotypes to that of HLS. All the fetuses and individuals reported by the authors showed lethal phenotypes consistent with a defect in hedgehog signaling and were similar to the phenotype described in the animal models of *KIAA0586*. SRTD with or without polydactyly refers to a group of skeletal ciliopathies with an autosomal recessive trait that is characterized by a constricted thoracic cage, short ribs, shortened tubular bones, polydactyly, and a ‘trident’ appearance of the acetabular roof [26]. Some forms of SRTD are lethal in the neonatal period due to respiratory insufficiency secondary to a severely restricted thoracic cage, whereas others are compatible with life [27]. Looking at all clinical descriptions, our case showed features shared in all three syndromes (Figure 1).

Overlapping features in genetic syndromes are increasing as we deepen our research on molecular diagnosis. At the same time, the number of new genetic disorders is also rising since new technologies are available to map and explore known genes that cause specific clinical phenotypes [28]. Although clinical and molecular diagnosis is crucial to elucidating an individual rare condition, addressing a possible variable expressivity of phenotypes with too many different names can be confusing and hinder the process more than aid. There are several explanations for variable expressivity and clinical heterogeneity in genomic disorders: genetic locus, variant type, number of genes involved, multiple allelism, protein isoforms, and modifier genes are some of them. In addition, a new theory of “true oligogenic” was also raised to understand such complexity: authors have proposed that the actions of two or more recessive genes with heterozygous mutations may result in a phenotype only when the mutations coexist (which may be our index case) [3,28,29].

Our patient showed a paternal inherited variant on *KIAA0586*, however, after an exome panel investigation for ciliopathies, we did not succeed in completely unraveling the molecular disarray. As the majority of ciliopathies are autosomal recessive inheritance, a heterozygous mutation would not fulfill the requirements to express the syndrome. Curiously, the proband mother had an intronic truncated variant on the same gene that could provide the missing “other half”. Recent studies reported cases of individuals diagnosed with ciliopathies that have both a heterozygous variant on an exon and a heterozygous variant on an intron, which justified the phenotype [30,31,32]. In these cases, the intronic variants led to a cryptic exon that somehow disrupted the encoded protein. Therefore, since exonic and intronic variants can be causative of ciliopathies, we believe that despite the known exon inherited variant, the aberrant intronic variant may have been inherited from the mother, leading to the clinical hydrolethalus phenotype. The abnormal intronic region would, then, replace the expected second exonic mutation. Although the intronic variant hypothesis could be a plausible response, we cannot assess the proband molecular diagnosis. Furthermore, the involvement of other genes or variants cannot be discarded.

## 4. Materials and Methods

### Molecular Analysis

High-resolution GTG banding karyotype analysis was performed from lymphocyte cell cultures (46, XX). DNA was isolated from peripheral blood lymphocytes using the Gentra Puregene Blood Kit (Qiagen, Valencia, CA, USA). The germline copy number variations (CNVs) were investigated using a high-density CytoScan HD Array platform (Affymetrix, Santa Clara, CA, USA) and the final data were analyzed by the Chromosome Analysis Suite software v.3.1 (Affymetrix, NetAffx Annotation Files version 33.1—hg19). A new generations sequencing (NGS) panel for ciliopathies (Cildag V1 panel) was performed in both the patient and parent’s samples using the SureSelect 174 genes kit (Agilent, Santa Clara, CA, USA) and the NextSeq500 sequencer (Illumina, San Diego, CA, USA). Gene coordinates cited throughout the manuscript were based on the genome build GRCh38/hg38.

The medical ethics committee of Universidade Federal de Ciências da Saúde de Porto Alegre and Hospital Santa Casa de Misericórdia de Porto Alegre approved the study (69178217.7.0000.5345). The study was conducted in accordance with the Helsinki declaration.

## 5. Conclusions

To sum up, as the number of ciliopathy-associated genes increases and the range of overlapping features between ciliopathies becomes more common, the need to better understand the genotype–phenotype correlation becomes clearer. In addition, elucidating the variable expressivity is essential to comprehending how many different syndromes we have. To achieve that, NGS panels may have to start including screening of intronic regions since the current scenario involves mostly molecular analysis focused on exploring exons. Overall, phenotype severity and molecular diagnosis should be investigated together to better propose a possible degree of ciliopathies disorders.

## Data Availability

All data generated or analyzed during this study are included in this article. Further inquiries can be directed to the corresponding author.

## Supplementary Materials

The supporting information can be downloaded at: https://www.mdpi.com/article/10.3390/ijms25147900/s1.
